# Measuring Emotion in Parliamentary Debates with Automated Textual Analysis

**DOI:** 10.1371/journal.pone.0168843

**Published:** 2016-12-22

**Authors:** Ludovic Rheault, Kaspar Beelen, Christopher Cochrane, Graeme Hirst

**Affiliations:** 1 Department of Political Science, University of Toronto, Toronto, Canada; 2 Informatics Institute, University of Amsterdam, Amsterdam, Netherlands; 3 Department of Computer Science, University of Toronto, Toronto, Canada; University of Pécs Medical School, HUNGARY

## Abstract

An impressive breadth of interdisciplinary research suggests that emotions have an influence on human behavior. Nonetheless, we still know very little about the emotional states of those actors whose daily decisions have a lasting impact on our societies: politicians in parliament. We address this question by making use of methods of natural language processing and a digitized corpus of text data spanning a century of parliamentary debates in the United Kingdom. We use this approach to examine changes in aggregate levels of emotional polarity in the British parliament, and to test a hypothesis about the emotional response of politicians to economic recessions. Our findings suggest that, contrary to popular belief, the mood of politicians has become more positive during the past decades, and that variations in emotional polarity can be predicted by the state of the national economy.

## Introduction

Our main goal in this paper is to adapt affective computing methods to the study of political discourse. We develop a methodology to produce domain-specific polarity lexicons and implement this approach using the entire corpus of proceedings of the British House of Commons during the past one hundred years. Next, our paper illustrates the potential of this methodology by tackling a specific question about the emotional states of policy-makers. We argue that politicians not only represent the preferences of their constituents over issues debated in parliament, they also react emotionally to national and world events in a manner that is predictable. In essence, politicians mirror the feelings and apprehensions of civilians in the face of adversity. We test this claim by tracking down the dynamics of politicians’ emotional responses during economic hard times. Our empirical results contribute to mounting evidence accumulated in social sciences about the linkages between emotion and human behavior [1–4].

Our decision to focus on emotions in political discourse was propelled by the rise in importance of two influential streams of literature. The first one concerns the psychology of human behavior. Since at least the 1960s, developments in the field of behavioral economics have brought the psychological aspects of decision-making to the forefront. Seminal works on bounded rationality [5], prospect theory [6, 7] and regret theory [8] have all attempted to tackle the observed discrepancies between theories grounded in rationality and actual human behavior. Lending additional credence to this field of research, a recent body of work stressed the conclusion that the emotionality of human decision-making has an intrinsic, neural basis [9–12]. The progress of neuroscience has even led some scholars to coin the term “neuroeconomics” to speak of its applications in the discipline of economics [13]. Implications for the study of political behavior have been surveyed in [3]. A primary concern behind this study is that making sense of the decisions made by elected politicians entails being able to tap into, in one way or another, their emotions. Until now, addressing this question has been hindered by the apparent difficulty of monitoring politicians from afar, let alone measuring their states of mind. The theory and methods that we introduce in this paper are an attempt to fill that gap, by focusing on large amounts of officially recorded political writings and by employing the practices developed in natural language processing.

The second stream of literature stems from computer science and concerns the detection of sentiment and emotion in textual data. More specifically, *affective computing* refers to a wide range of computational tools for the measurement of emotional states and affective responses communicated by humans either through facial and corporal expressions, oral or written speech [14–18]. These methods are sometimes referred to as emotional prosody or textual affect sensing. Recent work in this field has examined a variety of topics, from the use of emotions in musical lyrics [19] to the spread of happiness in social media [20, 21] and the study of medical conditions such as depression in online communities [22–24]. The digitization of book collections has also opened the door to linguistic research on a massive scale [25], which includes studies examining the expression of emotions in English literature during the past centuries [26–28]. To our knowledge, however, such methodologies had not been implemented on historical corpora of parliamentary debates until now.

Of particular relevance for this study are the prior attempts to apply affective computing methods for the study of political and socio-economic topics. Bollen et al., for instance, were able to measure meaningful emotional reactions among Twitter users in the face of real-world events such as the 2008 U.S. presidential campaign, shifts in market indicators of the recession and changes in oil prices [29]. Variations in the public mood as a response to economic and political events have also been studied in a growing number of papers (see e.g. [30, 31]). Another recent study has looked at the association between economic indicators and the polarity of words expressed in books during the last century [32]. The researchers found a significant relation between the misery index and the emotional tone of books published in the decade that followed: changes in economic conditions appear to be reflected in the authors’ use of language. This body of work provides evidence that individuals react emotionally to macroeconomic conditions, justifying further our interest in finding out whether politicians exhibit similar attitudes inside parliamentary institutions.

## Materials and Methods

Our corpus consists of all available volumes of the British House of Commons’ Hansard between 1909 and 2013 inclusive. It contains all the debates, oral questions and oral answers to written questions. The format of the Hansard—the official text archives of debates and speeches—was modified in 1909, when new standards were implemented for the verbatim record of the debates [33]. This is why our corpus begins at that date. Those text documents have been stored using a markup language following linked open data standards as part of the international project *Digging into Linked Parliamentary Data* (Dilipad). The corpus comprises a total of approximately 956.8 million tokens (i.e. words in a broader sense, including digits and other types of strings), with an average of 9.1 million tokens each year and a standard deviation of 2.2 million tokens. Considering the lemmatized version of the corpus—that is, the roots of words, which avoids duplicate counting of plural and conjugated verb forms—and considering only tokens appearing 10 times or more, UK parliamentarians have used a total vocabulary of 108,506 tokens. The length of parliamentary sessions has increased over time, and so has the corpus size per year: the decade 1910–1919 had an average annual size of 6.9 million tokens, compared to 9.8 million during the decade 2000–2009.

Many approaches have been developed for capturing emotions in textual data, using either machine learning classifiers trained on human-annotated corpora or lexicons (dictionaries), that is, lists of words associated with emotions. One of the main challenges with those approaches is that they can rarely be used across domains. Sentiment analysis classifiers trained with corpora from a specific domain were shown to have limited exportability to texts using a different register or genre [34–36]. A model trained on social media blurbs written in casual English would fare poorly if applied to parliamentary speeches, more sophisticated and restricted by the general decorum of political institutions. On the other hand, polarity lexicons are often more general, but they still have limitations when applied to corpora across domains. Popular examples of such lexicons include the NRC Word-Emotion Association Lexicon [37], in which words were annotated for eight specific emotions and positive/negative polarity using crowdsourcing, SentiWordNet [38], created using recursive algorithms based on the WordNet database, and the polarity lexicon of OpinionFinder [39]. (Other general-purpose lexicons for affective computing include the General Inquirer [40], the Linguistic Inquiry and Word Count (LIWC) dictionaries [41], Hu and Liu’s Opinion Lexicon [42], and WordNet extensions such as WordNet-Affect [43] and Q-WordNet [44].)

Like classifiers trained with non-political corpora, these lexicons are not tailored to the analysis of political speeches. Parliaments are associated with expressions that convey specific meanings and interpretations that we need to take into account. For instance, the first three lexicons mentioned above give a negative score to a word such as *war*, and positive ones to *education* and *health*. Yet, a word like *war* will inevitably be used more frequently in times of war, since the topic needs to be discussed in parliament. Assuming that debates become more negative simply because of the increased presence of this word would be misleading. Similarly, nouns like *education* and *health* have different meanings in politics as they relate to policy domains. They also identify specific departments and ministerial functions. An increased usage of the word *health* would provide little information about the tone of the debates taking place in the House of Commons, as this could merely reflect the presence of a bill about that specific issue on the agenda. In short, we would like to avoid attributing an emotional value to words without considering the fact that they may have a descriptive, domain-specific usage.

### Creating Domain-Specific Lexicons for Affective Computing

To overcome these problems, we rely upon a methodology for textual affect sensing that is adaptive to the domain under study. The general approach that we follow here has been introduced in [45] and a related methodology is discussed in [46]. We start by creating the vector space model of our corpus using the *GloVe* algorithm [47]. This model converts the vocabulary of our corpus into numerical vectors based on the matrix of word-word co-occurrences. We compute word vectors of 300 dimensions for each combination of lemma and part of speech (e.g. nouns, adjectives, and so forth), using a symmetric context window of 15 tokens. For simplicity, we use the expression “lemma” in what follows to speak of a lemma/part-of-speech pair. The second step of our methodology consists of creating an initial list of 200 seed lemmas capturing positive and negative emotions in the English language (100 lemmas for each pole). We selected these lemmas individually to ensure that they do not have multiple, opposite meanings when used as a specific part of speech, and to exclude terms with domain-specific meanings. Our objective is to use this list of seed lemmas as a starting point for the creation of emotion lexicons adaptive to any domain, given the availability of a large corpus. We provide additional details about these steps of our methodology in S1 Appendix.

Specifically, using vector distances to detect word similarities, we attribute to all other lemmas in the vocabulary a score indicating how close they are to each of the two groups of seeds. The formula corresponds to:

$$\begin{array}{c} s_{i}=\sum_{p=1}^{P}\frac{v_{i}\cdot v_{p}}{∥v_{i}∥∥v_{p}∥}-\sum_{q=1}^{Q}\frac{v_{i}\cdot v_{q}}{∥v_{i}∥∥v_{q}∥} \end{array}$$ (1)

where ‖**v**_*i*_‖ is the norm of vector **v**_*i*_ associated with lemma *i*, and where the seed lemmas for positive and negative emotions are indexed by *p* = {1, …, *P*} and *q* = {1, …, *Q*}, respectively. The scores *s*_*i*_ are scaled into a [−1, 1] interval reflecting their emotional polarity. We retain the 2000 lemmas with the highest and lowest scores, expanding our lexicon to 4200 words. To illustrate the output of this method, we report the first 20 lemmas with the highest and lowest scores in Table 1. By redistributing those scores to the lemmas across the original corpus, we are able to quantify the mood of parliamentary debates over time, which can be aggregated by session, month, quarter, or year.

**Table 1:** Highest Scores in Domain-Specific Polarity Lexicon.

Positive			Negative		
Lemma	PoS	*s*_*i*_	Lemma	PoS	*s*_*i*_
congratulate	verb	1.00	compound	verb	−1.00
delighted	adjective	0.96	dreadful	adjective	−0.96
high-quality	adjective	0.96	cause	verb	−0.96
tribute	noun	0.96	appalling	adjective	−0.92
commend	verb	0.92	grievous	adjective	−0.92
impressive	adjective	0.91	frightful	adjective	−0.88
worthwhile	adjective	0.91	inexcusable	adjective	−0.87
informative	adjective	0.89	exacerbate	verb	−0.80
enable	verb	0.89	ghastly	adjective	−0.80
warm	adjective	0.89	inflict	verb	−0.80

The table reports the first twenty positive and negative lemmas/part of speech (PoS) pairs in our adaptive polarity lexicon, along with their scores.

For the purpose of this study, we create a measure of emotional polarity as follows. (In the remainder of this text, we use the expression “emotional polarity” or “polarity” to speak of the aggregate indicator of emotional words in the British parliament. Concept usage varies across disciplines, and the term “sentiment” is also common to speak of positive and negative emotions in computer science.) Let *w*_*it*_ denote lemmas occurring in the text during period *t*. Next, let **1**{*w*_*i*_ ∈ *L*} be an indicator function equaling 1 if the lemma *w*_*i*_ is present in our lexicon, denoted by *L*, and 0 otherwise. Multiplying this function with the polarity score *s*_*i*_ associated with each lemma *i*, and summing over all lemmas occurring in a time period *t*, gives an absolute indicator of the emotional polarity of language during that period. We divide by the unweighted count of lemmas during each period, so that accurate comparisons over time are possible. This means computing

$$\begin{array}{c} \frac{\sum_{i=1}^{n_{t}}1\left\{w_{it}\in L\right\}s_{i}}{\sum_{i=1}^{n_{t}}1\left\{w_{it}\in L\right\}}, \end{array}$$ (2)

where *n*_*t*_ is the total number of lemmas in the Hansard during period *t*.

We improve this indicator further by accounting for the location of each lemma within negative sentences. We constructed a parameter, denoted *θ*_*it*_, measuring the valence of lemma *i* in the speeches of period *t*. This parameter is set to 1 unless *w*_*it*_ is located between a word indicative of a negative clause and a punctuation mark, in which case it equals 0 (words indicating negative clauses include *not*, *no*, *never*, *neither* and *nor*). Thereby, we avoid attributing positive scores to an expression such as “not satisfied”. Letting *y*_*t*_ denote the emotional polarity in the House of Commons at time *t*, our measure amounts to

$$\begin{array}{c} y_{t}=\frac{\sum_{i=1}^{n_{t}}1\left\{w_{it}\in L\right\}s_{i}\theta_{it}}{\sum_{i=1}^{n_{t}}1\left\{w_{it}\in L\right\}}. \end{array}$$ (3)

Higher values of *y*_*t*_ indicate more positive debates. The measure of polarity, just like the score variable *s*_*i*_, can be negative or positive. However, positive words are used more frequently in the English language and as a result, aggregate measures will tend to remain in the positive range as the corpus length increases. Our interest lies in the temporal change in *y*_*t*_; thus, the scaling of that measure is irrelevant. Importantly, by using the count of lemmas as the denominator in Eq (3), we account for the fact that parliamentary sessions may differ in length from one period to the next, and avoid biases caused by the overall frequency of positive words.

We assessed the substantive validity of our measures and included more detailed information on this stage in S1 Appendix. In particular, we evaluated our approach against a corpus of film reviews commonly used to assess polarity classifiers, in which users’ self-reported scores can be considered a basis for ground validity. We found that our measure of emotional polarity alone can predict up to 80% of film ratings accurately, and classifiers incorporating our indicator as a feature compare favorably to other models for sentiment analysis in the literature. We should point out, however, that our approach does not account for the full structure of sentences. Utterances that our method may not capture easily are complex uses of language such as sarcasm, irony and hyperbole. On the other hand, notice that we rely on word vectors designed to capture the underlying meaning of terms in the Hansard vocabulary. Thus, a positive word commonly used to express the opposite of a speaker’s true feelings is likely to be found in a negative context across the corpus, and the resulting scores would capture that semantic ambiguity. A rather straightforward extension to the methodology presented here could also model vectors for entire phrases or sentences, instead of lemmas, using tools for latent semantic analysis such as singular value decomposition [48] or document vectors [49]. For the purpose of this study, we believe that the benefits and simplicity of our approach outweigh its limitations.

### Emotional Polarity Trends in the British Parliament

**Fig 1:**
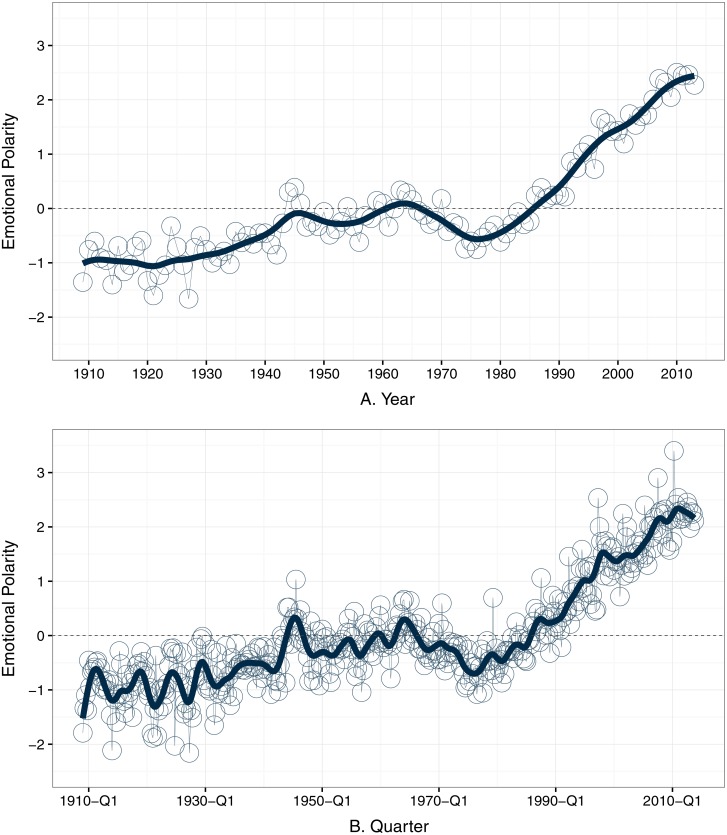
Emotional Polarity in the British House of Commons, 1909–2013.

Fig 1 depicts our measures of polarity over time along with smoothing splines, computed by year and by quarter. These measures have been normalized as standard scores; thus negative values indicate yearly or quarterly scores below the sample mean. Arguably the most striking feature of these graphs is the clear rising trend in both indicators over time, suggesting that political debates have become more positive in recent years. This last observation may sound counterintuitive for contemporary observers of political affairs. Conventional wisdom suggests that politics has become more negative in recent years, although existing research on the tone of political discourse has focused mostly on peripheral evidence. For instance, some have documented a negative tone in the news coverage of politics [50] or a decline in public support for governments [51]. Our results show that the British parliament, on the contrary, has become more positive than it used to be. In fact, the trends appear consistent with the major turns of events of the past century. The first two decades of our sample mark an era of negative polarity in the British House of Commons, in line with the social divide of those turbulent years. The 1910s and 1920s were punctuated by major labor disputes, including a nation-wide strike in 1926, the rise of the workers’ movement and the perceived threat of socialism with the arrival into power of a new Labour Party in 1924. Adding to that were the First World War and the Irish War of Independence of 1919–1921. The current tone of parliamentary proceedings seems to be far more optimistic compared to the early 20th Century, in line with the relative peace and stability characterizing the more recent decades (see [52]).

**Fig 2:**
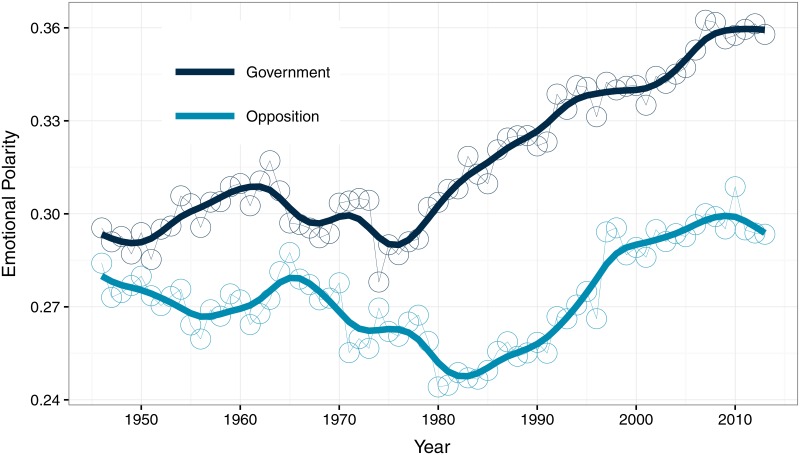
Emotional Polarity of Government and Opposition in Britain, 1946-2013.

We examined this trend in a number of ways to assess its robustness. We first compared the time-series obtained using a number of alternative polarity lexicons. The finding of debates becoming more positive over time resurfaces even when considering indicators based on the three general lexicons mentioned above (see S1 Fig). We also investigated changes in usage for the 200 initial seed lemmas considered to construct our domain-specific lexicon. We found that the relative usage of our negative seed words has actually increased slightly over time, based on data from the *Google Books Ngram Viewer*. The average relative frequency of our 100 positive seeds has decreased by 9 percent from the decade 1909–1918 to the decade 1999–2008, whereas the average for our 100 negative seeds has increased by 2 percent over the same period. This suggests that the pattern we observed in Fig 1 is not simply derived from the choice of specific seeds to create our lexicon. To investigate further, we tested whether the trend in emotional polarity is explained by an increasing gap between the tone of speeches of members from the party forming the government on one hand, and members of the opposition on the other hand (that is, members of the parliament (MPs) not affiliated with the party in power). We naturally expect the government to be more positive than the opposition, whose members have the duty to question the party in power and challenge its decisions [53]. Notice that we call those groups of MPs “government” and “opposition” for short, even though the government—the executive branch—is the Cabinet. Since Westminster countries are characterized by strong party discipline, it is unlikely that MPs from the party in power would criticize the government, which is why we group them together. When making the distinction between these parliamentary functions, we still find increasing trends in our polarity indicator (Fig 2). Although the party in power remained more positive than the opposition—which is unsurprising—the upward trend of the last few decades appears to generalize to the House of Commons as a whole.

Our yearly and quarterly indicators also exhibit the characteristics of long-memory processes, as suggested by the slow decay of their autocorrelation functions (ACF) (S2 Fig). This long-memory feature means that exogenous shocks affecting the mood of politicians can have a lasting effect on the nature of debates over time. The power spectra of the series are reported in S3 Fig. Using linear regressions on a log scale, we estimated parameters from power spectral densities of the form 1/*f*^*α*^, where *f* is the frequency and *α* a parameter which should approximate 1 in the presence of pink noise. The fitted value of *α* is close to 1 when using the quarterly series (1.03, with a 95% confidence interval of [0.86, 1.20]). This parallels a number of other societal processes that have previously been found to be heavy-tailed [54]. Since our measure is the result of a large number of micro-interactions between members of parliament with shared histories, the finding that mood spreads over time following a 1/*f* process is not surprising. We note, however, that the parameter *α* in the yearly series is closer to 2 (1.71, with a 95% confidence interval of [1.38, 2.04]), a characteristic of Brownian noise. This has implications for the choice of estimators in the rest of our analysis, as Brownian noise is associated with unit root processes [55]. Accordingly, we tested each time-series for stationarity. Tables A-D in S1 Appendix report the full results. The main measures of emotional polarity used in our empirical analysis below appear to follow a unit root process, based on several specifications of both the Dickey-Fuller and KPSS tests.

## Empirical Results

As explained at the outset, we are interested in explaining changes in emotional polarity over time. In other words, we are considering an equation of motion in discrete time of the type

$$\begin{array}{c} \Delta y_{t}=f\left(\Delta y_{t-1},x_{t}\right), \end{array}$$ (4)

where Δ*y*_*t*_ is the first-difference operator of our polarity measure, and *x*_*t*_ is a measure of national events affecting the mood of politicians. Specifically, we expect that the mood in parliament will respond to economic business cycles, that is, the core periodic transitions between economic recessions and expansions. Our theoretical argument is that those business cycles are a fundamental force affecting most spheres of activity in a polity; hence, recessions are likely to trigger a large amount of stress on workers and businesses that should realistically have repercussions in the House of Commons. The mandate of elected politicians is to represent their constituents. We usually think of this representation in terms of positions to be expressed on issues. In our view however, the emotions of civilians are just as likely to be echoed inside political institutions. This can take the form of harsher questions directed at the government in power and heightened discord during debates over bills and motions. In short, we expect the mood to become negative during recessions, as opposed to periods of economic expansion. Below, we test this expectation using both a binary indicator of recessions and continuous indicators of the national economy.

We begin with the computation of autoregressive models including two exogenous components. The first is a simple binary indicator *r*_*t*_ equaling 1 for years encompassing a recession, where a recession is defined as two or more consecutive quarters of negative growth in the gross domestic product (GDP), and 0 otherwise. For the time-period where quarterly data are missing, we coded a year as 1 if GDP growth was negative during that year. The full list of recessions appears in S1 Appendix, Table E. In addition to economic cycles, we control for the occurrence of political cycles caused by the periodicity of democratic institutions. We include a second binary indicator *e*_*t*_ equaling 1 if a general election has been held during a given year, and 0 otherwise. We considered various other empirical indicators as control variables, in particular binary measures accounting for wars and the party in power, but those turn out to be poor predictors of emotional polarity and have little impact on our results (see S1 Appendix). For the sake of parsimony, we focus on the simpler models here. Our empirical specification can be expressed in terms of the autoregressive-moving average framework including explanatory factors treated as exogenous (ARMAX). The model corresponds to:

$$\Delta y_{t}=\alpha_{0}+\beta_{1}r_{t}+\beta_{2}e_{t}+\sum_{i=1}^{l}\rho_{i}\left(\Delta y_{t-i}-\alpha_{0}-\beta_{1}r_{t-i}-\beta_{2}e_{t-i}\right)+\varepsilon_{t},$$ (5)

where *l* is the lag length and *ε*_*t*_ an error term. The first-difference transformation produces a stationary *y*_*t*_ series, and our estimators satisfy the usual stability conditions. We report maximum likelihood estimates computed with one and two lags in Table 2. The estimated autoregressive parameters are negative, indicating that random shocks to the rate of change in emotional polarity (*y*_*t*_) eventually vanish following oscillatory decays. The estimated coefficient for the Recession variable is negative, which is consistent with our expectation that the mood in parliament responds negatively to economic downturns. The value of the coefficient –0.198 in the first model indicates that a recession is associated with a 0.2 point decrease in the annual change in polarity. The estimate is statistically significant at the 95% confidence level in specifications with 1 and 2 autoregressive lags. Conversely, election years appear to increase the positivity of the mood by a similar order of magnitude (0.19). We also computed mean difference tests (*t*-tests) by considering bivariate relationships one at a time. Table 3 reports the average differences in polarity for recession years, election years, and years at war. Once again, the difference associated with recessions is negative and statistically significant.

**Table 2:** Autoregressive Models of Polarity in UK Parliament.

Δ*y*_*t*_	Model 1	Model 2
Recession	−0.198 (0.062)	−0.165 (0.063)
Election	0.187 (0.069)	0.183 (0.069)
Intercept	0.034 (0.032)	0.027 (0.030)
*ρ*_1_	−0.275 (0.095)	−0.313 (0.098)
*ρ*_2_		−0.145 (0.104)
N	104	104
Log-Likelihood	−21.156	−20.214
AIC	52.311	52.428
BIC	65.533	68.294

Autoregressive models of the change in emotional polarity (Δ*y*_*t*_), with Recession (*r*_*t*_) and Election (*e*_*t*_) included as binary exogenous variables. Standard errors are reported in parentheses.

**Table 3:** Emotional Polarity, the Economic Cycle, Elections and Wars.

	Obs.	Δ*y*_*t*_	*t*	*p*-value
Recession = 0	83	0.083		
Recession = 1	21	−0.136		
Difference	104	−0.219	−2.895	0.002
Election = 0	79	−0.014		
Election = 1	25	0.188		
Difference	104	0.202	2.735	0.004
Wars = 0	82	0.034		
Wars = 1	22	0.037		
Difference	104	0.002	0.027	0.511

One-sided *t*-tests of mean differences in the annual first differences Δ*y*_*t*_, by group, along with *p*-values.

### Continuous Indicators of Economic Conditions

For the next step, we investigate the robustness of the relationship between national economy and political mood by considering continuous indicators. We selected four pertinent annual time series available for the entire time period: a measure of labor disputes (the natural logarithm of the number of days lost due to strikes per year), the rate of unemployment, the misery index (the sum of unemployment and inflation), and the rate of growth of the gross domestic product (GDP). The latter may not represent an ideal measure from a scientific standpoint since it merely reflects variations in the size of an economy, rather than its wealth—measures of economic growth in the field of economics are usually per capita indicators, to account for changes in the size of the population. In practice, however, policy-makers routinely rely upon the growth in the GDP to assess whether a country faces a recession or to establish targets. As a result, we expect this particular indicator to be relevant for the British House of Commons. Obviously, those indicators are strongly related to each other, but they capture different dimensions of economic cycles. Unemployment and the misery index are, arguably, more likely to affect constituents in their daily life and as a result, to trigger a response from elected officials. Labor disputes can be viewed as an even more concrete measure of public discontent regarding economic conditions. The idea that labor disputes can be a relevant indicator to model the mood of politicians should make sense for most observers of politics. Strikes and related labor conflicts are disruptive social activities that are fundamentally political, as well as emotionally laden for the actors involved.

**Fig 3:**
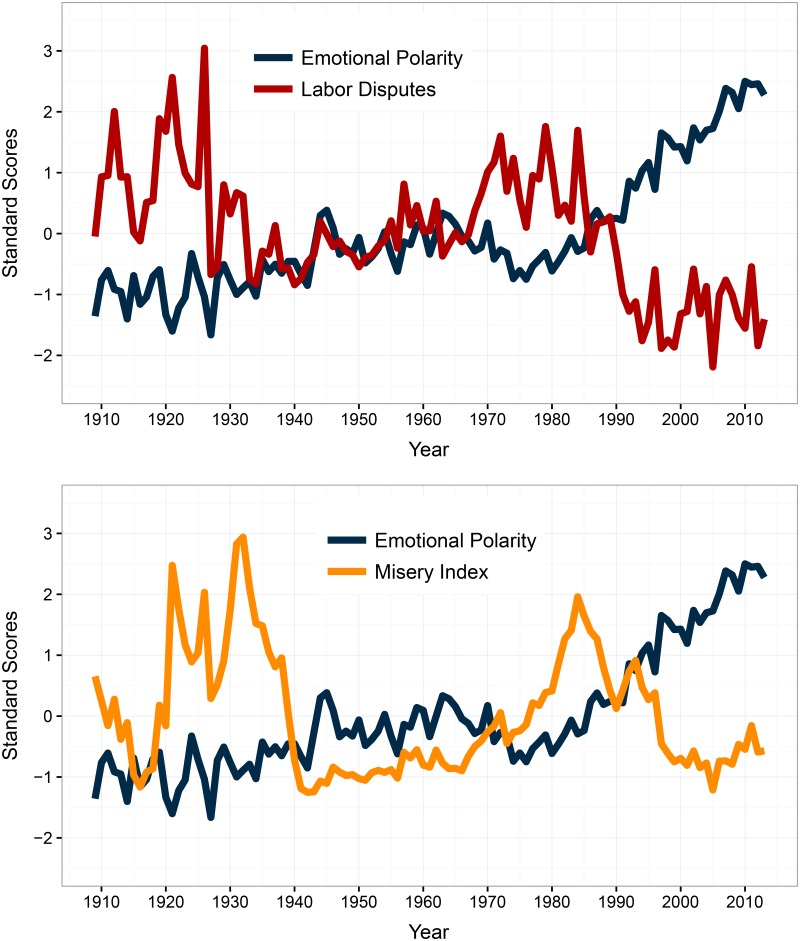
The Emotional Polarity of British Politicians and the National Economy.

Although we found signs of a statistical relation between each of these four measures and our indicator of emotional polarity, the labor disputes and misery index series exhibit the clearest association. Both series are superimposed on the emotional polarity indicator in Fig 3. As can be seen, the intensity of labor disputes and the misery index appear counter-cyclical to the polarity of debates in the parliament. In particular, the major recession of 1973–1975 matches a peak in the negativity of debates, a surge in labor conflicts and an unprecedented spike in the level of the misery index caused by soaring inflation. The five key indicators mentioned so far are also plotted using a heat map in S4 Fig. These figures are useful to grasp the big picture of the last century in Britain. The early 20th Century was characterized by intense labor disputes and skyrocketing rates of unemployment, culminating with the Great Depression of the 1930s. Things changed drastically after the second World War, when most of these measures stabilized. Meanwhile, the mood of parliamentary debates became more positive. A second period of turbulence arose in the 1970s and 1980s, decades encompassing two important recessions and new episodes of intense work conflicts. Accordingly, the mood became increasingly negative, before experiencing an upward trend in the recent years. On the other hand, the period encompassing the Second World War seems to shift away from that pattern. The end of the war coincides with relatively positive speeches in parliament, while the country was in fact struggling economically after the prolonged war effort. We explain this discrepancy, however, by the political significance of the victory for Britain. The war period also witnessed a series of coalition governments in the British parliament, which limited opportunities for partisan clashes on the usual issues of contention.

**Fig 4:**
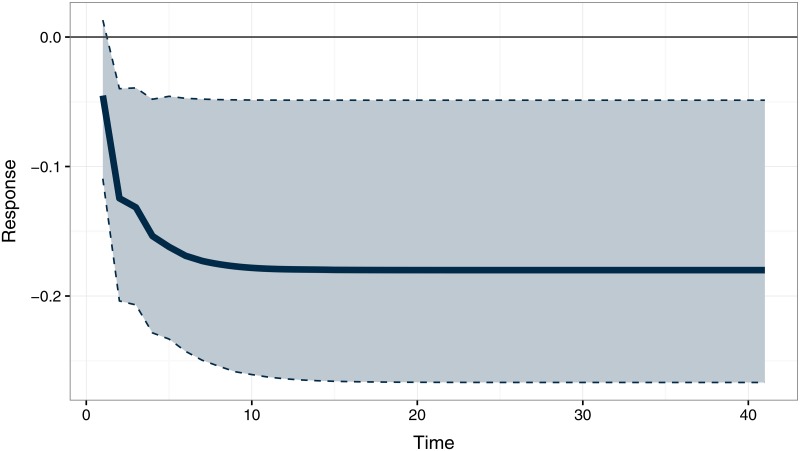
The Effect of Labor Disputes on Emotional Polarity. Orthogonalized impulse response function with bootstrapped error bands computed from a bivariate VECMs with an unrestricted constant and 2 lags in levels. The plot illustrates the estimated response of polarity to a one standard deviation shock in the labor disputes series.

Comparing figures visually does not give a definitive idea of the statistical association between these variables. For this reason we perform pairwise Granger causality tests between emotional polarity and these four historical measures. As can be seen in Table 4, one relationship in particular is revealed to be Granger-causal: the one going from labor disputes to emotional polarity. Since both variables are unit root processes, we tested for the existence of a cointegrating vector using the Johansen rank test. We find evidence of cointegration (see Table F in S1 Appendix), which suggests that the temporal trajectories followed by the mood of parliamentarians and the intensity of labor disputes are tied together. We estimated this relation using a vector error correction model (VECM). The fitted cointegrated vector corresponds to

$$y_{t}=-0.06-1.39x_{t}$$ (6)

where *x*_*t*_ is the indicator of labor disputes. Eq (6) suggests that, in the long run, both series tend to stay negatively related, more than a standard deviation apart. Since the variables are moving as a system, this should not be interpreted as a causal effect. One way to assess the dynamics between the number of strikes and the polarity of speeches in the House of Commons is using an orthogonalized impulse response function computed from the VECM estimates (Fig 4). The emotional response to a standard deviation shock in the labor disputes series is negative, converging to a value of approximately –0.18. The series being non-stationary, any random shock is persistent, and so is the estimated effect. Or, put another way, the estimated effect of a labor conflict persists until the initial shock is offset by another shock in the opposite direction.

**Table 4:** Granger Causality Tests.

Cause	Effect	*χ*^2^	d.f.	Pr > *χ*^2^
Labor Disputes	Polarity	9.424	1	0.002
Polarity	Labor Disputes	0.056	1	0.813
Unemployment	Polarity	0.471	1	0.493
Polarity	Unemployment	2.401	1	0.121
Misery Index	Polarity	0.223	1	0.637
Polarity	Misery Index	1.441	1	0.230
GDP Growth	Polarity	0.168	1	0.682
Polarity	GDP Growth	0.689	1	0.406
Labor Disputes	Polarity–Government	0.092	1	0.761
Polarity–Government	Labor Disputes	0.001	1	0.977
Labor Disputes	Polarity–Opposition	8.048	1	0.005
Polarity–Opposition	Labor Disputes	0.006	1	0.939

The table shows tests of the null of Granger non-causality for integrated processes based on augmented pairwise VAR models.

We examined the robustness of this finding using alternative estimators such as a dynamic ordinary least squares (DOLS). Table G in S1 Appendix reports the full set of estimates. This estimator is also suitable for modeling cointegrated relations by assuming the direction of causality, and may be easier to interpret. Using emotional polarity as the endogenous variable, the estimated long-run effect of labor disputes is approximately –0.9, and statistically significant at the 99.9% confidence level using heteroskedasticity and autocorrelation consistent standard errors. In other words, a one standard deviation increase in the levels of labor disputes is associated with a nearly proportional decrease, around 0.9 standard deviation, in the normalized indicator of the polarity of speeches in the British parliament. Together, these results suggest a strong statistical relationship between the two series.

Finally, making the distinction between party in power and opposition helps to identify the causal mechanism at work. The bottom part of Table 4 tests the causality of labor disputes for each group of parliamentarians. As can be seen, occurrences of labor conflicts Granger-cause the emotional polarity of opposition parties in the House of Commons, but not that of the party in power. To assess this result, we estimated the emotional response of each group of MPs to a shock in labor disputes, again with VECMs. Using either the yearly or quarterly dataset, the size of the effect of labor disputes appears greater when considering opposition parties (see S5 Fig), which suggests that the emotional response of politicians transits via the opposition first and foremost. This finding lends additional support to our claim that political discourse reflects the mood of the electorate in meaningful ways. The opposition in British democratic institutions was designed to keep the executive accountable by making inquiries in the House of Commons [56]. Economic downturns or labor conflicts are by nature undesirable situations, and it makes sense to think that opposition parties will be the first ones to decry them. Parties in power face different incentives. Being ultimately accountable for the state of affairs in the country, government MPs are better off avoiding outright recognition of social ills whenever they can, which could otherwise undermine their popularity. Our findings help to interpret the inverse relationship observed in Fig 3: labor conflicts make the discourse of opposition parties more negative, which in turns affects the tone of speeches in the parliament as a whole.

## Conclusion

The method discussed in this paper to measure emotion in political discourse has several benefits. It is relatively simple to use, it can be applied to different domains as long as a sufficiently large corpus exists, and it allows scoring the emotional valence of lemmas on a continuous scale. In fact, it is possible to use this approach to generate lexicons measuring different features of language, not just emotional polarity, as long as researchers can provide seed words to serve as a basis for computing word similarities from the vector space models. Applying this method to the corpus of the British Hansard from 1909 to 2013 leads to insightful findings. In particular, we found that politicians’ usage of emotional words is significantly related to economic cycles, a result supported by models using both a binary indicator of recession and a more politicized indicator of labor conflicts.

The observation that debates have become more positive in tone during the last decades raises an interesting question for social science scholars. Does this trend reflect a more general transformation of attitudes in society, or is it limited to politics? Although expanding the analysis to multiple corpora is a task that fell beyond the scope of the present research, existing work can provide some insight. For instance, one recent article analyzed long-term trends in the Google Books database using affective computing methods, in particular with an indicator based on the difference between the frequency of words associated with joy and sadness [28]. The findings suggest that books published in the late 20th Century had a more positive tone than those released in the 1970s and the 1980s, which parallels the trajectory depicted in Fig 1. Another study mentioned in our introduction found an association between economic misery and the expression of negative emotions in books [32]. The authors argued that economic considerations have become part of the shared experiences that influenced the general culture during the past century. Connecting our findings with these other studies suggests that improvements in living conditions may cause positive mood swings that transcend the political realm.

On the other hand, our findings somewhat diverge from a body of literature interested in the economics of happiness. Whether higher incomes make individuals happier is a question that attracted intense scrutiny in the social sciences [57]. To be sure, there is empirical support to the conclusion that better living conditions are associated with higher self-reported levels of happiness in cross-sectional surveys [58–61]. However, scholars have often failed to observe rising aggregate levels of happiness in the long run, even as countries get richer [57, 62]. This paradox contrasts with our findings, which exhibit long-range dependence. The divergence may be related to measurement. Answers to close-ended survey questions at fixed points in time give only a limited range of options to measure the dynamics of emotional states, as opposed to a full text corpus recording the language of politicians on an almost daily basis. Alternatively, there may be meaningful differences in the way various publics react to changes in economic conditions, differences that we do not fully understand yet. Future studies could take advantage of the type of methods presented here as an alternative way to tap into people’s emotions, and advance our knowledge on that fundamental question.

The findings highlighted in this paper also point to other implications for future research. For instance, if politicians react emotionally to economic downturns, it matters to reassess whether these emotions have in turn an impact on crucial decisions made during those periods. The indicators that we proposed in this paper could be used to pursue fine-grained analyses of this type. Moreover, we found evidence that emotional polarity follows a long-memory process, which is consistent with earlier findings about many social phenomena. To examine the persistence of moods, the Hansard corpus could be used to test for the presence of emotional contagion in the parliamentary network, as was done previously using social media data [63]. We coped with this property of the indicators by considering empirical methods that can accommodate integrated processes, but additional research could provide more insights on this particular question. Overall, given the importance of legislation and the ripple of impacts it begets on societies, we believe that improving our comprehension of the factors that alter the mood of policy-makers is an important research objective for the social sciences.

## Supporting Information

S1 Fig Emotional Polarity in Britain using General-Purpose Lexicons. Alternative measures of emotional polarity computed using three popular sentiment lexicons that are not specific to the domain of parliamentary debates: NRC, OpinionFinder and SentiWordNet.
(EPS)

S2 Fig Autocorrelation Functions of Emotional Polarity Measures. (EPS)

S3 Fig Power Spectral Densities of Emotional Polarity Measures (Log Scale). (EPS)

S4 Fig Emotional Polarity and Economic Indicators in the United Kingdom. Heat map of the five main indicators, illustrating the change from lowest to highest values during the whole time-period.
(EPS)

S5 Fig The Effect of Labor Disputes on Emotional Polarity, by Government and Opposition Status. Orthogonalized impulse response functions with bootstrapped error bands computed from bivariate VECMs with unrestricted constants. Yearly models (upper part) are computed with 2 lags in levels and quarterly models (lower part) with 5 lags in levels. The left panels show the response of the polarity indicator for the government MPs to a one standard deviation shock in the labor disputes series. Panels on the right show the response of the polarity indicator for the opposition MPs to the same increase in the number of labor disputes.
(EPS)

S1 Appendix Supporting Information for “Measuring Emotion in Parliamentary Debates with Automated Textual Analysis”. (PDF)
