# Supplementary material for: Measuring Emotion in Parliamentary Debates with Automated Textual Analysis
Source: PLoS One. 2016 Dec 22;11(12):e0168843. doi: 10.1371/journal.pone.0168843 (PMC5179059; doi:10.1371/journal.pone.0168843)
Supplement: S1 Appendix — (PDF) [file pone.0168843.s006.pdf]

S1 Appendix. **Supporting Information for "Measuring Emotion in Parliamentary Debates with Automated Textual Analysis"**

Ludovic Rheault[1], Kaspar Beelen[2], Christopher Cochrane[1], Graeme Hirst[3]

[1]Department of Political Science, University of Toronto, Toronto, Canada
[2]Informatics Institute, University of Amsterdam, Amsterdam, Netherlands
[3]Department of Computer Science, University of Toronto, Toronto, Canada

Preprocessing of the Text and Construction of the Lexicon

Our corpus comes from three different sources. The Hansard for the period 1936–2013 was formatted using a markup language by the team of the Dilipad project (`http://dilipad.history.ac.uk/`), using the files previously processed by the independent project *They Work For You* (`http://www.theyworkforyou.com/`). We processed the remaining part of the Hansard from 1909 to 1935 using the digitized archives available on the website of the UK parliament (`http://www.hansard-archive.parliament.uk/`) and after collecting missing volumes from a different source, the Millbank Systems website, an official repository of the digitized Hansard (`http://hansard.millbanksystems.com/`). We cleaned the early files for structural mistakes such as broken sentences or irregular spacing, using a custom Python script. We also duplicated the years from 1936 to 1938 to confirm that the final corpora collected from these sources are virtually identical. A few volumes are missing from the online archives, and the final values of our quarterly measures were linearly interpolated to fill in three missing quarters. The digitization is of good quality, although the corpus is not entirely free of typographic errors, likely caused by the optical character recognition routines used to create the archive. Foreign words with accentuation are the most problematic and were excluded from our analysis.

The preparation of the Hansard corpus necessitates intensive computing tasks. We processed the corpus on a Dell PowerEdge R520 server with Intel Xeon E5-2470 2.3GHz processors (32 cores) and 96GB of RAM, although our scripts should be efficient enough to run on most computers. For the purpose of this study, the corpus was split into sentences, tokenized, part-of-speech tagged, and lemmatized using the Stanford CoreNLP library [1], which is written in Java and available freely to researchers (`http://nlp.stanford.edu/software/corenlp.shtml`). We computed the vector space model using the *GloVe* algorithm, the source code of which, written in C, is also available to researchers (`http://nlp.stanford.edu/projects/glove/`). As mentioned in the text, we created a model with 300 dimensions, considering co-occurrences up to 15 words to the left and to the right.

A sensitive part of our approach consists of selecting the initial seed words that will serve as a basis for the creation of a polarity lexicon. Tables H and I list all the seeds we used for this study. To ensure that our approach adapts to the specificity of each domain, the seeds ought to be very general words used to express positive and negative emotions. Our objective is to create a list of seeds broad enough so that the creation of scores is not driven only by a few individual words, yet small enough so that a large portion of the emotion lexicon can be adapted using the vector space model of the

corpus under study. Based on our inspection of the available words meeting our criteria, we determined that 100 seeds for each pole was a reasonable cutoff point.

We created the list of seeds manually using the following set of rules. We began by identifying core, basic words used to express positive and negative emotions in the English language. Those words (*good*, *love*, and *happy* for the positive pole; *bad*, *hate*, and *sad* for the negative pole) were then searched recursively for synonyms using two open-source dictionaries and thesauruses: the Collaborative International Dictionary of English and WordNet 3.0. After examining these synonyms individually, we retained words as seeds only if they respected the following rules:

1. Polysemous seed words need to have an unambiguous emotional orientation, which means that multiple meanings of the same word used as the same PoS must not have opposite polarities.

2. Seed words cannot be the name of an institution, parliamentary procedure or political topic (excluded are words such as *war*, *dispute*, *unemployment*, and so forth).

3. Seed words need to be basic and common words of everyday language.

Notice that since we distinguish between parts of speech, we may still include a word that has opposite polarities when used as a verb as opposed to a noun, for instance, by including the orientation-relevant word/PoS pair. Once a list of potential seeds was established, we reduced its size to 100 for both positive and negative terms by selecting the most frequent in the English language. To have an estimate of their frequency, we queried the Google Ngram database for the period 1909–2008. We report the relative frequencies along with each seed in Tables H and I.

We took a number of additional steps to prevent the contamination of our measures by the idiosyncrasies of parliamentary life. We removed all non-informative expressions used as formal epithets to address members of parliament, which are used frequently in the Hansard. These include expressions such as “My Honourable Friend”, “The Right Honourable Member”, and so forth. Members of parliament are required to use them by protocol, hence they cannot be associated with emotions. Virtually all instances of the word *honourable* left in the final corpus are used in an actual sentence, rather than being a form of speech required by the decorum of the House of Commons. We also removed all indicators of nationalities (e.g. *Americans*, *Czechoslovakian*, and so forth) as they should be theoretically neutral. To eliminate typos and rare words, we removed lemmas occurring less than 200 times in the corpus. Finally, we purged the corpus of all digits and proper nouns when computing polarity lexicons.

Assessing the Methodology

The methodology proposed to generate domain-relevant polarity lexicons responds to a number of concerns associated with the analysis of large corpora. This section describes the properties of this approach and reports on validity tests performed with a dataset of movie reviews commonly used in the literature on machine learning, for comparison purposes.

A key benefit of the methodology used in this paper is that it accounts for the evolution of language. Some expressions used at the beginning of the 20th Century may be infrequent in recent years, or have disappeared entirely. Moreover, some words may be highly period-specific, relating to issues of the day. References to the *Soviet Union*, for instance, are unlikely to occur in the House of Commons two decades after its dissolution, whereas *Russia* and its inflections should be more common. This would be problematic if someone attempted to classify speeches based on a model trained

during a specific period of time. The approach we use here alleviates these concerns. To begin with, we rely upon a vector space representation computed using the word-word co-occurrence matrix of the entire corpus, which spans over one century. This implies that emotional polarity scores depend on the aggregation of all possible word meanings over the entire period, accounting for changes that may have occurred in the usage of English. In particular, our approach is robust to meaning reversal: words associated with a negative (positive) connotation in the early 20th Century and a positive (negative) one in the 21st Century would be ranked as neutral, since the contradictory meanings and hence their occurrence with seed words will tend to offset each other. To further prevent any issues with the change in language over time, we purposely divided the Hansard corpus into three equal time-periods of 35 years, and considered words that appeared at least 10 times in all of the three periods. This way, we exclude the words whose usage has stopped, recent neologisms, and other words that are highly specific to an era or to a legislature.

We assessed the validity of our approach using a human-annotated corpus from a different domain, namely a corpus of 50,000 film reviews corresponding to the training and testing sets used in [2] to evaluate polarity classifiers. The reviews were originally extracted from the *Internet Movie Database* (IMDb) website, where users can write their own evaluations of films and rate them using a numerical scale ranging from 1 to 10. Thus, we can test our approach by assessing how well our measure of emotional polarity predicts the score given by users, on the ground that users who enjoyed a film are more likely to express positive emotions than users who hated it. The existence of previous studies using the same dataset also provides us with a benchmark for comparisons. The film reviews were annotated as positive if the user rated a film at 7 or higher, and negative if the film is rated 4 or lower. We created the domain-specific lexicon using a similar but larger corpus containing close to 7.9 million reviews extracted from the Amazon website and discussed in [3]. This larger corpus contains all sorts of product reviews, including books, music albums and films. Because of the similarity of purpose and usage, we expect reviews from the larger corpus to be closely similar in register and genre to those from the IMDb. Using a larger set of reviews to build our lexicon makes the co-occurrence matrix more accurate. The Amazon corpus being close in size to the British Hansard corpus, our validity tests are based on an approach that is comparable to that used in the rest of our analysis.

Table J reports accuracy measures computed using support vector machine (SVM) classifiers with a linear kernel. In all cases, we fit the SVMs with the training set of 25,000 reviews and use the estimates to predict the classes in the testing set, also containing 25,000 reviews. We start by considering the performance of our polarity indicator alone. Our measure is constructed as defined in the main text, although instead of time periods, we compute an emotional polarity score for each film review. With this variable alone as a predictor, the accuracy of classification reaches close to 75%. We also consider a subset of reviews with "extreme ratings", that is, film reviews rated as either 1 or 10, converted into a binary variable. Accuracy exceeds 80% when classifying those extreme reviews. Next, we combine our polarity measure to other word features, namely the term-frequency/inverse document frequency (TF-IDF) weighted document term matrix—a so-called "Bag-of-Words" (BoW) model. This increases the accuracy of prediction to 88.3 and 92.4%, respectively for the main 25,000 reviews in the testing set and the subset of extreme reviews only. These levels of accuracy are at least as good as several of the benchmark results presented along with the original study that introduced the dataset [2, Table 2], where the highest level of accuracy is 88.9%, for a BoW model including additional features.

We further tested models that account for film features, which may interfere with the sentiments of reviewers. Indeed, genres that feature darker themes (e.g. horror,

drama, film noir) may have reviews more negative in tone simply because they include descriptive statements about the film, rather than expressing the emotions of users. Thus, a better machine learning model would control for specific film genres. An even stronger predictor of individual user ratings is the average score for a given film, computed from the ratings of all users of the website. A SVM classifier including all these features reaches a predictive accuracy of 89.5 and 93.3%, respectively for the full testing set and the subset of extreme reviews. As for the proportional reduction in errors, it ranges from 49 to 87% across the models. Since our interest lies in the capacity of measuring variations in the emotional states of a speaker on a continuous scale, the accuracy of document classification tasks captures only imperfectly the purpose of our methodology. However, the clear relationship between our indicator of polarity and the ratings given to films by users brings a strong support to the idea that our lexicons are accurately tapping into human emotions expressed in writings.

Robustness Tests and Additional Results

Our empirical analyses are performed using the R programming language. All Granger causality tests are computed using the method proposed in [4], to account for the possibility of cointegrated relationships. The VECMs used for this study are of the form

$$\Delta \mathbf{z}_t = \boldsymbol{\alpha}(\boldsymbol{\beta}' \mathbf{z}_{t-1} + \boldsymbol{\mu}) + \sum_{i=1}^{l} \boldsymbol{\Pi}_i \Delta \mathbf{z}_{t-i} + \boldsymbol{\lambda} + \boldsymbol{\varepsilon}_t \qquad (1)$$

where $\mathbf{z}_t$ is a vector comprising labor disputes x_t and our indicator of emotional polarity y_t, $\boldsymbol{\alpha}$ is a vector of short-run adjustment parameters, $\boldsymbol{\beta}$ is a vector of cointegrating parameters and $\boldsymbol{\lambda}$ is a vector of intercepts capturing possible trends in the levels of the series. Models with additional exogenous variables such as the party in power (Labour or Conservative), elections or seasonal dummies were also tested. Since the results remain substantively the same, only the most parsimonious models are discussed in the text. Nevertheless, we present a number of alternative specifications below. When computing orthogonalized impulse responses, we set the ordering in the Cholesky decomposition as (x_t, y_t), from the hypothesized most exogenous series to the most endogenous.

In addition to the main results reported in the paper, we conducted a variety of robustness tests based on alternative specifications and empirical models. To begin, Table K reports ARIMAX models in which we check the robustness of the impact of recessions after controlling for other possible confounding factors. The first model includes an additional binary variable indicating whether Britain is at war in a given year. As can be seen, the inclusion of this variable leaves the original result practically unchanged; in fact, the new variable itself turns out to be statistically insignificant. The second model also includes a variable measuring which party is in power, either Labour (Party = 0) or Conservative (Party = 1). Since Britain experienced a prolonged period of national governments during which no single party formed the government, we restrict the sample to the period 1946–2013. In case of years during which both parties shared power due to a change of government, we coded the variable based on which party was in power for the largest portion of that year. As shown in Table K, the inclusion of this variable does not affect the main results discussed in the text, and once again it does not turn out to be a strong predictor of polarity.

Next, we also replicated our models using the quarterly version of the dataset, to the extent feasible. The quarterly dataset comprises more observations, but the limited availability of economic data in quarterly format for the full 20th Century prevents us from fully exploiting our polarity indicator and restricts the time-period

covered. The results shown in Table L are a replication of the autoregressive models presented in the empirical section of the main text. We considered a multiplicative seasonal ARIMAX model with three autoregressive (AR) lags and two seasonal AR lags. The specification was chosen based on information criteria and corresponds to an ARIMAX$(3,0,0) \times (2,0,0)_4$. Only the polarity indicator is first-differenced (labeled Δy_t in the text). Note that as before, we do not difference the binary variables *Recession*, *Election* and *Wars*. First, there is no statistical justification for it, these series being not unit root processes. Second, this would modify the theoretical implications by focusing only on the change from one state to another (i.e. the impact of the beginning and the end of a recession, rather than the impact of the recession itself). The results reported in Table L parallel those presented earlier, based on the yearly data. The estimated short-run coefficient measuring the impact of a quarter of recession on the change in emotional polarity is about -0.07, which means a 0.07 reduction in the rate of change of MPs' emotional polarity. Given that recessions last more than one quarter—they have an average duration of 3.5 quarters in our sample—the total impact of a recession requires adding the effects together. Taking into account the change in time units, our estimates turn out to be close in magnitude to those obtained using yearly data. The effect of a recession is not statistically significant in the last model considered in Table L, although still of the expected sign. We also report unit root tests based on quarterly data in Tables C and D, for completeness. The outcomes of these tests are generally similar to those reported using the yearly data. Overall, these additional results suggest that our main findings are, for the most part, robust to changes in the periodicity of the data.

Finally, Tables N and O report the full results of the VECMs discussed in the main paper, respectively using yearly and quarterly data. Those estimates must be assessed with care since the models are meant to be interpreted as a system, which is why we report the effects using impulse responses in S5 Fig. However, the adjustment parameters in $\boldsymbol{\alpha}$ can be informative. They measure the responsiveness of a variable to a shock in the system, and values indistinguishable from zero can be interpreted as a sign of weak exogeneity. For instance, the estimates in the middle column and top portion of Table N suggest that the emotional polarity of the government is weakly exogenous in the relationship with labor disputes, since the adjustment parameter is non-significant. In contrast, the polarity of the opposition is endogenous (last column). This is consistent with the interpretation proposed in the main text.

Data Sources for Economic and Political Variables

1. Real *Gross Domestic Product* (GDP) corresponds to a chained series in millions of pounds with the reference year 2011, as compiled in the dataset "Three Centuries of Data" published by the Bank of England and retrieved on February 1, 2015. The series is described in detail in [5]. It covers the period 1909–2013.

   The quarterly version of the indicator is also taken from [5], and covers the periods from 1920–Q2 to 1939–Q4 and from 1955–Q2 to 2013–Q4.

2. The *Recession* variable is measured by coding values as 1 when a given year encompasses a sequence of two or more quarters with negative growth in the real GDP variable, and 0 otherwise. When quarterly data are missing, the year is coded 1 if the annual rate of growth of real GDP is negative, zero otherwise. For the quarterly data, the variable equals 1 for every quarter of a recession and zero otherwise, where a recession is a sequence of two or more consecutive quarters of negative GDP growth. The construction of this indicator is detailed in Table E.

3. The *Election* variable equals 1 if at least one general election was held in a given year (some years had more than one general election), and 0 otherwise. For the quarterly dataset, the variable equals 1 if a general election was held in a given quarter, and 0 otherwise.

4. The *Wars* variable equals 1 if the United Kingdom is engaged in a major armed conflict in a given year (or quarter), and zero otherwise. The list of relevant wars or conflicts considered is presented in Table P.

5. The *Labor Disputes* indicator measures the number of days lost due to labor disputes in the United Kingdom per year. The series is taken from the Labour Market Statistics Dataset published by the Office for National Statistics (ONS) of the United Kingdom, released on April 17, 2015. It corresponds to the series labeled BBFW. The quarterly version of the indicator is taken from the same source, and is available from 1931–Q1 to 2013–Q4.

6. The *Unemployment* rate is taken from the "Three Centuries of Data" dataset for the period 1909–2013. The quarterly version of the indicator corresponds to the harmonized unemployment rate and is taken from the OECD's Main Economic Indicators database, series LMUNRRTT, seasonally adjusted. The series cover the period from 1955–Q1 to 2013–Q4.

7. *Inflation* (required to compute the *Misery Index*) is computed from the rate of growth of the Consumer Price Index, extracted from the series CDSI in the Consumer Price Inflation time series dataset (MM23) published by the ONS, and retrieved on May 25, 2016. The Misery Index is obtained by taking the sum of inflation and unemployment.

   The quarterly version of the inflation indicator is the quarter-on-quarter rate of growth in the consumer price index (all items), retrieved from OECD's Main Economic Indicators database, series CPALTT01. The quarterly growth series covers the period from 1955–Q2 to 2013–Q4. The Misery Index is obtained by taking the sum of inflation and of the seasonally adjusted rate of unemployment.

Scripts

Table Q is a summary of the scripts and programs used to process the British Hansard corpora, identifying the purpose of each script and the programming language used. The languages were selected for their speed and suitability for particular tasks. The scripts and datasets are available online at `https://github.com/lrheault/emotion`.

Tables

Table A. ADF Unit Root Tests, Yearly Data.

Series	Model	1 Lag	2 Lags	95% c.v.	Outcome
Polarity	Drift	–0.506	0.120	–2.88	Non-Stationary
Polarity	Trend	–2.435	–1.869	–3.43	Non-Stationary
Polarity (Government)	Drift	–0.340	–0.239	–2.89	Non-Stationary
Polarity (Government)	Trend	–2.032	–1.918	–3.45	Non-Stationary
Polarity (Opposition)	Drift	–1.247	–0.981	–2.89	Non-Stationary
Polarity (Opposition)	Trend	–1.719	–1.494	–3.45	Non-Stationary
Labor Disputes (Log)	Drift	–2.572	–2.156	–2.88	Non-Stationary
Labor Disputes (Log)	Trend	–3.385	–2.865	–3.43	Non-Stationary
Unemployment	Drift	–2.450	–2.711	–2.88	Non-Stationary
Unemployment	Trend	–2.438	–2.702	–3.43	Non-Stationary
Misery Index	Drift	–2.319	–2.611	–2.88	Non-Stationary
Misery Index	Trend	–2.372	–2.708	–3.43	Non-Stationary
GDP Growth	Drift	–5.567	–5.875	–2.88	Stationary
GDP Growth	Trend	–5.582	–5.922	–3.43	Stationary

Augmented Dickey-Fuller (ADF) t-test statistics of the null of unit roots based on annual time series, using 1 and 2 lags. The results compare models with a drift and models with a linear trend.

Table B. KPSS Unit Root Tests, Yearly Data.

Series	Test Statistic	Value	95% c.v.	Lags	Outcome
Polarity	μ	1.635	0.463	4	Non-Stationary
Polarity	τ	0.317	0.146	4	Non-Stationary
Polarity (Government)	μ	1.511	0.463	3	Non-Stationary
Polarity (Government)	τ	0.325	0.146	3	Non-Stationary
Polarity (Opposition)	μ	0.561	0.463	3	Non-Stationary
Polarity (Opposition)	τ	0.353	0.146	3	Non-Stationary
Labor Disputes (Log)	μ	0.849	0.463	4	Non-Stationary
Labor Disputes (Log)	τ	0.200	0.146	4	Non-Stationary
Unemployment	μ	0.144	0.463	4	Stationary
Unemployment	τ	0.143	0.146	4	Stationary
Misery Index	μ	0.210	0.463	4	Stationary
Misery Index	τ	0.134	0.146	4	Stationary
GDP Growth	μ	0.123	0.463	4	Stationary
GDP Growth	τ	0.071	0.146	4	Stationary

Kwiatkowski-Phillips-Schmidt-Shin (KPSS) tests of the null of stationarity, using annual time series. Lag length is selected automatically using the formula $4(T/100)^{1/4}$, where T is the sample size.

Table C. ADF Unit Root Tests, Quarterly Data.

Series		Model 4 lags	5 lags	95% c.v.	Outcome
Polarity	Drift	–1.123	–0.998	–2.87	Non-Stationary
Polarity	Trend	–3.100	–2.948	–3.42	Non-Stationary
Polarity (Government)	Drift	–0.785	–0.722	–2.87	Non-Stationary
Polarity (Government)	Trend	–2.922	–2.723	–3.42	Non-Stationary
Polarity (Opposition)	Drift	–1.954	–1.965	–2.87	Non-Stationary
Polarity (Opposition)	Trend	–2.525	–2.506	–3.42	Non-Stationary
Labor Disputes	Drift	–2.605	–2.142	–2.87	Non-Stationary
Labor Disputes	Trend	–3.047	–2.623	–3.42	Non-Stationary
Unemployment	Drift	–1.859	–1.650	–2.88	Non-Stationary
Unemployment	Trend	–1.648	–1.410	–3.43	Non-Stationary
Misery Index	Drift	–1.499	–5.197	–2.88	Non-Stationary
Misery Index	Trend	–1.312	–5.363	–3.43	Non-Stationary
GDP Growth	Drift	–6.030	–2.073	–2.88	Stationary
GDP Growth	Trend	–6.177	–1.875	–3.43	Stationary

Augmented Dickey-Fuller (ADF) t-test statistics of the null of unit roots based on quarterly time series, with 4 and 5 lags. The results compare models with a drift and models with a linear trend.

Table D. KPSS Unit Root Tests, Quarterly Data.

Series	Test Statistic	Value	95% c.v.	Lags	Outcome
Polarity	μ	5.043	0.463	5	Non-Stationary
Polarity	τ	0.881	0.146	5	Non-Stationary
Polarity (Government)	μ	3.817	0.463	5	Non-Stationary
Polarity (Government)	τ	0.755	0.146	5	Non-Stationary
Polarity (Opposition)	μ	1.358	0.463	5	Non-Stationary
Polarity (Opposition)	τ	0.853	0.146	5	Non-Stationary
Labor Disputes	μ	1.754	0.463	5	Non-Stationary
Labor Disputes	τ	0.965	0.146	5	Non-Stationary
Unemployment	μ	1.411	0.463	4	Non-Stationary
Unemployment	τ	0.786	0.146	4	Non-Stationary
Misery Index	μ	1.172	0.463	4	Non-Stationary
Misery Index	τ	0.955	0.146	4	Non-Stationary
GDP Growth	μ	0.200	0.463	4	Stationary
GDP Growth	τ	0.046	0.146	4	Stationary

Kwiatkowski-Phillips-Schmidt-Shin (KPSS) tests of the null of stationarity using quarterly time series. Lag length is selected automatically using the formula $4(T/100)^{1/4}$, where T is the sample size.

Table E. Recessions in the United Kingdom, 1909–2013.

Years	Quarters	Notes
1917		Negative Annual GDP Growth
1919		Negative Annual GDP Growth
1920, 1921	1920–Q3 to 1921–Q2	
1926	1926–Q2 to 1926–Q3	
1930, 1931	1930–Q2 to 1931–Q3	Great Depression
1932	1932–Q2 to 1932–Q3	Great Depression
1944, 1945, 1946, 1947		Post-WW2 Depression, Negative Annual GDP Growth
1956	1956–Q2 to 1956–Q3	
1961	1961–Q3 to 1961–Q4	
1973, 1974	1973–Q3 to 1974–Q1	
1975	1975–Q2 to 1975–Q3	
1980, 1981	1980–Q1 to 1981–Q1	
1990, 1991	1990–Q3 to 1991–Q3	
2008, 2009	2008–Q2 to 2009–Q2	

List of years coded as recessions, along with the corresponding sequences of two or more consecutive quarters of negative GDP growth in the sample. When quarterly data is not available, years are considered in recession if there is negative GDP growth in that year as a whole.

Table F. Johansen Cointegration Tests, Yearly Data.

Cointegration Rank	Lags	Trace Stat.	Lags	Trace Stat.	95% c.v.
Labor Disputes, Polarity					
H_0: Rank = 0	1	41.661	2	20.649	15.41
H_0: Rank = 1	1	1.259	2	0.298	3.76
Labor Disputes, Polarity (Government)					
H_0: Rank = 0	1	17.471	2	10.365	15.41
H_0: Rank = 1	1	0.492	2	0.022	3.76
Labor Disputes, Polarity (Opposition)					
H_0: Rank = 0	1	25.837	2	13.730	15.41
H_0: Rank = 1	1	2.888	2	1.797	3.76

The table reports trace statistics of the Johansen cointegration rank tests. All variables have been normalized. The labor disputes series has been previously transformed on the natural log scale. The tests are computed using an unrestricted constant (i.e. with a linear trend in the undifferenced series). A rejection of the null hypothesis of a rank of 0 indicates the presence of a cointegrating relationship.

Table G. Dynamic Ordinary Least Squares (DOLS), Yearly Data.

	Leads and Lags					
**Emotional Polarity**	**1**	**2**	**1**	**2**	**1**	**2**
Labor Disputes	–0.881<br>(0.187)	–0.902<br>(0.179)				
Misery Index			–0.368<br>(0.216)	–0.374<br>(0.218)		
Unemployment					–0.122<br>(0.179)	–0.122<br>(0.185)
Intercept	0.015<br>(0.166)	0.001<br>(0.152)	–0.002<br>(0.307)	–0.021<br>(0.283)	–0.001<br>(0.338)	–0.018<br>(0.326)
Observations	102	100	102	100	102	100
Adjusted R^2	0.595	0.600	0.092	0.077	–0.015	–0.033
σ	0.621	0.603	0.931	0.916	0.984	0.969

Dynamic Ordinary Least Squares (DOLS) models with the main polarity indicator as the dependent variable. Included are specifications for which both series are integrated. The models include lags and leads of first differences of the right-hand side variables in the regression, with the lag length indicated in the column headers. Heteroskedasticity and autocorrelation consistent standard errors are reported in parentheses. σ is the standard deviation of the residuals (the standard error of the regression).

Table H. Positive Seed Lemmas by Google Ngram Relative Frequency.

Lemma	PoS	Frequency	Lemma	PoS	Frequency
well	adv.	0.715	wonderful	adj.	0.031
good	adj.	0.555	friendly	adj.	0.030
important	adj.	0.337	pleasant	adj.	0.029
best	adj.	0.261	creative	adj.	0.028
better	adj.	0.243	worthy	adj.	0.027
true	adj.	0.234	friendship	noun	0.026
love	verb	0.205	sympathy	noun	0.026
able	adj.	0.192	nice	adj.	0.025
help	verb	0.188	honour	noun	0.025
strong	adj.	0.147	comfort	noun	0.025
solution	noun	0.138	honest	adj.	0.024
importance	noun	0.129	genuine	adj.	0.024
respect	noun	0.123	healthy	adj.	0.024
truth	noun	0.115	intelligent	adj.	0.023
strength	noun	0.101	welcome	adj.	0.023
effective	adj.	0.099	helpful	adj.	0.023
success	noun	0.099	encourage	verb	0.022
freedom	noun	0.092	praise	noun	0.022
significant	adj.	0.091	dignity	noun	0.021
interesting	adj.	0.084	prosperity	noun	0.021
useful	adj.	0.078	comfortable	adj.	0.020
successful	adj.	0.075	reliable	adj.	0.019
beautiful	adj.	0.073	succeed	verb	0.019
appropriate	adj.	0.068	delight	noun	0.019
fair	adj.	0.067	merit	noun	0.018
happy	adj.	0.059	lovely	adj.	0.018
perfect	adj.	0.058	splendid	adj.	0.018
gain	verb	0.055	sympathetic	adj.	0.017
excellent	adj.	0.053	generous	adj.	0.017
superior	adj.	0.051	vigorous	adj.	0.017
fairly	adv.	0.050	perfection	noun	0.017
reasonable	adj.	0.050	appreciate	verb	0.016
secure	verb	0.049	loving	adj.	0.016
efficiency	noun	0.049	magnificent	adj.	0.016
valuable	adj.	0.049	integrity	noun	0.015
properly	adv.	0.047	talent	noun	0.015
improvement	noun	0.046	kindly	adv.	0.015
safe	adj.	0.043	fortunately	adv.	0.014
desirable	adj.	0.039	grateful	adj.	0.014
satisfactory	adj.	0.039	glorious	adj.	0.013
wise	adj.	0.039	fortunate	adj.	0.013
protect	verb	0.038	clever	adj.	0.012
truly	adv.	0.036	sincere	adj.	0.012
satisfaction	noun	0.036	confident	adj.	0.012
efficient	adj.	0.035	delightful	adj.	0.012
joy	noun	0.035	strengthen	verb	0.011
improve	verb	0.033	respected	adj.	0.011
enjoy	verb	0.032	admirable	adj.	0.010
happiness	noun	0.031	smart	adj.	0.009
glad	adj.	0.031	satisfying	adj.	0.009

The table shows the positive seed lemmas/part-of-speech (PoS) pairs used to create the domain-specific lexicon, along with the Google Ngram frequency of each lemma, per thousand words, averaged over years between 1909 and 2008. Frequencies were retrieved from the database on April 9, 2015.

Table I. Negative Seed Lemmas by Google Ngram Relative Frequency.

Lemma	PoS	Frequency	Lemma	PoS	Frequency
problem	noun	0.223	hate	verb	0.017
death	noun	0.216	complaint	noun	0.017
difficult	adj.	0.142	painful	adj.	0.017
loss	noun	0.116	worry	verb	0.017
bad	adj.	0.089	unfortunate	adj.	0.017
fear	noun	0.085	neglect	verb	0.016
failure	noun	0.079	prejudice	noun	0.015
enemy	noun	0.071	disaster	noun	0.015
wrong	adj.	0.068	distress	noun	0.015
difficulty	noun	0.068	hatred	noun	0.014
pain	noun	0.065	tragic	adj.	0.014
ill	adj.	0.063	shame	noun	0.014
risk	noun	0.062	breach	noun	0.013
danger	noun	0.060	contempt	noun	0.013
error	noun	0.057	unhappy	adj.	0.013
evil	adj.	0.054	frightened	adj.	0.013
criticism	noun	0.047	regret	noun	0.013
false	adj.	0.046	corruption	noun	0.013
weak	adj.	0.041	restriction	noun	0.012
dangerous	adj.	0.041	poorly	adv.	0.011
excess	noun	0.040	fraud	noun	0.010
damage	noun	0.040	miserable	adj.	0.010
lose	verb	0.038	stupid	adj.	0.010
worse	adj.	0.037	injustice	noun	0.010
afraid	adj.	0.036	ugly	adj.	0.010
fail	verb	0.034	wicked	adj.	0.010
sick	adj.	0.033	disadvantage	noun	0.009
unfortunately	adv.	0.030	disappointment	noun	0.009
confusion	noun	0.029	unfair	adj.	0.009
burden	noun	0.029	nonsense	noun	0.009
anxiety	noun	0.028	ridiculous	adj.	0.009
terrible	adj.	0.027	undesirable	adj.	0.009
suffer	verb	0.027	imperfect	adj.	0.009
fault	noun	0.026	harmful	adj.	0.009
anxious	adj.	0.026	horrible	adj.	0.009
destroy	verb	0.025	disastrous	adj.	0.008
worst	adj.	0.025	unsatisfactory	adj.	0.008
excessive	adj.	0.025	hopeless	adj.	0.008
threat	noun	0.025	complain	verb	0.008
mistake	noun	0.025	fearful	adj.	0.008
inferior	adj.	0.023	unjust	adj.	0.008
weakness	noun	0.023	irrelevant	adj.	0.008
anger	noun	0.022	corrupt	adj.	0.008
hurt	verb	0.022	unreasonable	adj.	0.008
angry	adj.	0.021	restrict	verb	0.007
tragedy	noun	0.020	careless	adj.	0.007
abuse	noun	0.020	grim	adj.	0.007
inadequate	adj.	0.020	wretched	adj.	0.007
sad	adj.	0.020	discomfort	noun	0.007
harm	verb	0.020	brutal	adj.	0.006

The table shows the negative seed lemmas/part-of-speech (PoS) pairs used to create the domain-specific lexicon, along with the Google Ngram frequency of each lemma, per thousand words, averaged over years between 1909 and 2008. Frequencies were retrieved from the database on April 9, 2015.

Table J. Accuracy Tests on Human-Annotated Film Reviews.

Features	Accuracy (%)	PRE (%)	F1 Score	N
Full Testing Set				
Polarity Only	74.6	49.1	0.739	25,000
Polarity + BoW	88.3	76.6	0.882	25,000
Polarity + BoW + Film Features	89.5	79.0	0.895	25,000
Extreme Reviews Only				
Polarity Only	80.7	61.3	0.798	10,021
Polarity + BoW	92.4	84.8	0.922	10,021
Polarity + BoW + Film Features	93.3	86.5	0.932	10,021

Accuracy and goodness-of-fit measures from support vector machine classifiers. Accuracy is the percentage of reviews correctly predicted and PRE is the proportional reduction in errors, or the percentage reduction in errors compared to a null model using the mode as the predicted category. BoW stands for the Bag-of-Words set of features computed with a term-frequency/inverse document frequency (TF-IDF) weighting scheme. Each model is computed with binary classes for positive and negative reviews. See S1 Appendix for a full description of each model.

Table K. Autoregressive Models of Polarity in UK Parliament, Yearly Data: Alternative Specifications.

Δy_t	**Model 3**	**Model 4**	**Model 5**	**Model 6**
Recession	–0.204<br>(0.062)	–0.174<br>(0.063)	–0.202<br>(0.069)	–0.182<br>(0.067)
Election	0.198<br>(0.069)	0.193<br>(0.070)	0.051<br>(0.076)	0.047<br>(0.078)
Wars	0.064<br>(0.061)	0.051<br>(0.058)	0.081<br>(0.099)	0.068<br>(0.097)
Party			0.062<br>(0.050)	0.064<br>(0.043)
Intercept	0.019<br>(0.035)	0.016<br>(0.032)	0.012<br>(0.048)	0.011<br>(0.043)
ρ_1	–0.290<br>(0.095)	–0.325<br>(0.098)	–0.330<br>(0.118)	–0.393<br>(0.126)
ρ_2		–0.134<br>(0.105)		–0.176<br>0.124
Observations	104	104	68	68
Log-Likelihood	–20.619	–19.830	–2.565	–1.577
AIC	53.537	53.659	19.130	19.154
BIC	69.104	72.170	34.666	36.910

Alternative specifications of time-series autoregressive models of the change in emotional polarity (Δy_t) in the UK Parliament, with Recession (r_t), Election (e_t), Wars and Party in power included as binary exogenous regressors. Standard errors are reported in parentheses.

Table L. Autoregressive Models of Polarity in UK Parliament, Quarterly Data.

Δy_t	**Model 1**	**Model 2**	**Model 3**
Recession	–0.073<br>(0.027)	–0.077<br>(0.026)	–0.048<br>(0.033)
Election	0.162<br>(0.069)	0.172<br>(0.069)	0.287<br>(0.096)
Wars		0.056<br>(0.039)	0.105<br>(0.052)
Party			0.023<br>(0.015)
Intercept	0.008<br>(0.008)	0.005<br>(0.008)	–0.021<br>(0.014)
ρ_1	–0.830<br>(0.055)	–0.834<br>(0.054)	–0.786<br>(0.066)
ρ_2	–0.645<br>(0.074)	–0.648<br>(0.073)	–0.609<br>(0.095)
ρ_3	–0.495<br>(0.081)	–0.500<br>(0.080)	–0.401<br>(0.108)
θ_1	–0.243<br>(0.088)	–0.249<br>(0.088)	–0.211<br>(0.113)
θ_2	–0.129<br>(0.071)	–0.132<br>(0.071)	–0.167<br>(0.076)
Observations	310	310	235
Log-Likelihood	–145.515	–144.462	–93.421
AIC	309.030	308.924	208.842
BIC	342.659	346.290	246.898

Time-series autoregressive models of the change in emotional polarity (Δy_t) in the UK Parliament using quarterly data. Standard errors are reported in parentheses. Regular autoregressive components are denoted ρ_i whereas seasonal autoregressive components are denoted θ_i. The sample is non-contiguous due to the limited availability of quarterly GDP data, covering the periods from 1920–Q3 to 1938–Q4 and from 1955–Q3 to 2013-Q4.

Table M. Johansen Cointegration Tests, Quarterly Data.

Cointegration Rank	Lags	Trace Stat.	Lags	Trace Stat.	95% c.v.
Labor Disputes, Polarity					
H_0: Rank = 0	5	22.524	6	18.081	15.41
H_0: Rank = 1	5	0.464	6	0.394	3.76
Labor Disputes, Polarity (Government)					
H_0: Rank = 0	5	18.574	6	14.366	15.41
H_0: Rank = 1	5	0.404	6	0.428	3.76
Labor Disputes, Polarity (Opposition)					
H_0: Rank = 0	5	25.641	6	24.164	15.41
H_0: Rank = 1	5	2.451	6	2.259	3.76

The table reports trace statistics of the Johansen cointegration rank tests computed with 4 and 5 lags in first differences (5 and 6 in the levels of the series). All variables have been normalized. The labor disputes series has been previously transformed on the natural log scale. The tests are computed using an unrestricted constant (i.e. with a linear trend in the undifferenced series). Rejection of the null hypothesis of a rank of 0 indicates the presence of a cointegrating relationship.

Table N. Vector Error Correction Models, Yearly Data.

	Full Sample	Party in Power	Opposition Parties
Emotional Polarity Equation (Δy_t)			
Adjustment Parameter	–0.096<br>(0.036)	–0.034<br>(0.048)	–0.221<br>–0.068
Δx_{t-1}	–0.011<br>(0.049)	0.060<br>(0.064)	0.006<br>–0.102
Δy_{t-1}	–0.203<br>(0.092)	–0.249<br>(0.125)	–0.219<br>–0.110
Constant	0.043<br>(0.031)	0.059<br>(0.036)	0.009<br>–0.052
Labor Disputes Equation (Δx_t)			
Adjustment Parameter	–0.256<br>(0.076)	–0.274<br>(0.090)	–0.060<br>–0.092
Δx_{t-1}	–0.196<br>(0.104)	–0.269<br>(0.120)	–0.382<br>–0.136
Δy_{t-1}	0.199<br>(0.196)	0.267<br>(0.233)	0.028<br>–0.147
Constant	–0.016<br>(0.066)	–0.007<br>(0.066)	-0.033<br>–0.070
Cointegrating parameter (Normalized as $1y_t = \mu + \hat{\beta}x_t$)			
$\hat{\beta}$	–1.393<br>(0.204)	–1.205<br>(0.256)	–1.252<br>(0.252)
Observations	103	66	66
R^2 (Δx_t)	0.151	0.113	0.236
R^2 (Δy_t)	0.246	0.294	0.191

The table reports the full output of vector error correction models (VECMs) computed with one lag in first differences (two lags in levels) and an unrestricted constant (a trend in the levels of the series). The emotional polarity indicator is based on the parliamentary groups indicated in the column headers. Since this is a system estimator, the coefficients in this table should not be interpreted individually; refer to the text for interpretation. Impulse responses estimated from the second and third models are reported in S5 Fig(A) and (B).

Table O. Vector Error Correction Models, Quarterly Data.

	**Full Sample**	**Party in Power**	**Opposition Parties**
Emotional Polarity Equation (Δy_t)			
Adjustment Parameter	–0.065<br>(0.027)	–0.035<br>(0.030)	–0.206<br>(0.050)
Δx_{t-1}	0.047<br>(0.041)	0.003<br>(0.041)	0.181<br>(0.065)
Δx_{t-2}	0.017<br>(0.040)	–0.019<br>(0.040)	0.107<br>(0.064)
Δx_{t-3}	0.075<br>(0.038)	0.051<br>(0.038)	0.137<br>(0.060)
Δx_{t-4}	0.071<br>(0.033)	0.041<br>(0.033)	0.105<br>(0.053)
Δy_{t-1}	–0.686<br>(0.059)	–0.634<br>(0.067)	–0.536<br>(0.069)
Δy_{t-2}	–0.519<br>(0.068)	–0.381<br>(0.076)	–0.452<br>(0.074)
Δy_{t-3}	–0.297<br>(0.066)	–0.171<br>(0.075)	–0.226<br>(0.071)
Δy_{t-4}	–0.028<br>(0.054)	0.009<br>(0.062)	–0.003<br>(0.060)
Constant	0.027<br>(0.021)	0.023<br>(0.021)	0.007<br>(0.034)
Labor Disputes Equation (Δx_t)			
Adjustment Parameter	–0.177<br>(0.046)	–0.223<br>(0.057)	–0.120<br>(0.060)
Δx_{t-1}	–0.392<br>(0.070)	–0.367<br>(0.078)	–0.464<br>(0.078)
Δx_{t-2}	–0.215<br>(0.068)	–0.212<br>(0.076)	–0.286<br>(0.077)
Δx_{t-3}	–0.225<br>(0.064)	–0.200<br>(0.072)	–0.262<br>(0.073)
Δx_{t-4}	–0.145<br>(0.056)	–0.150<br>(0.062)	–0.192<br>(0.063)
Δy_{t-1}	0.112<br>(0.100)	0.191<br>(0.127)	0.072<br>(0.083)
Δy_{t-2}	0.120<br>(0.115)	0.207<br>(0.145)	0.012<br>(0.090)
Δy_{t-3}	0.208<br>(0.112)	0.284<br>(0.142)	0.067<br>(0.086)
Δy_{t-4}	0.184<br>(0.093)	0.299<br>(0.118)	0.092<br>(0.072)
Constant	–0.010<br>(0.035)	–0.004<br>(0.040)	–0.012<br>(0.041)
Cointegrating parameter (Normalized as $1y_t = \mu + \beta x_t$)			
$\hat{\beta}$	1.281<br>(0.190)	1.180<br>(0.177)	1.021<br>(0.153)
Observations	327	269	269
R^2 (Δy_t)	0.393	0.327	0.388
R^2 (Δx_t)	0.302	0.314	0.280

The table reports the full output of vector error correction models (VECMs) computed with four lags in first differences (five lags in the levels of the series) and an unrestricted constant (a trend in the level variables). The emotional polarity indicator is based on the parliamentary groups indicated in the column headers. Since this is a system estimator, the coefficients in this table should not be interpreted individually; refer to the text for interpretation. Impulse responses estimated from the second and third models are reported in S5 Fig(C) and (D).

Table P. Major Wars and Armed Conflicts Involving the United Kingdom, 1909–2013.

Years	Quarters	War
1914–1918	1914–Q3 to 1918–Q4	World War I
1919–1921	1919–Q1 to 1921–Q3	Irish War of Independence
1939–1945	1939–Q3 to 1945–Q2	World War II
1950–1953	1950–Q2 to 1953–Q3	Korean War
1969	1969–Q3	The Troubles (Northern Ireland)
1972	1972–Q3	The Troubles (Northern Ireland)
1982	1982–Q2	Falklands War
2003	2003–Q1 to 2003–Q2	Iraq War (Operation Iraqi Freedom)
2006–2007	2006–Q1 to 2007–Q2	Afghanistan War (Southern Afghanistan operations)

List of years and quarters coded as being at war, including intrastate armed conflicts, based on substantive knowledge about the relevance of those armed conflicts for the United Kingdom. For prolonged conflicts, only the most intense periods are included.

Table Q. Summary of Scripts.

Name	Language	Description
early-hansard-parser.py	Python 2.7	A script to parse XML files of the early Hansard volumes from the UK Parliament.
millbank-scraper.py	Python 2.7	A script to scrape the Millbank Systems website and retrieve Hansard volumes missing from the UK Parliament archives.
modern-hansard-parser.py	Python 2.7	A script to parse XML files of the modern Hansard (post 1936), in the Political Mashup format.
CoNLLSetup.class	Java 8	A custom class to use the Stanford CoreNLP library.
remove-decorum-words.sh	Bash	A Perl-based Shell script to remove expressions required by the decorum of the House (e.g. "The Right Honourable").
valence-shifter.R, looper.so	C, R 3.2	An R wrapper to add a valence-shifting variable to the CoNLL corpus, using C for speed.
lexicon-generator.R	R 3.2	An R script to generate domain-specific lexicons based on the word vectors obtained using the Glove program.
lexicon-join.py	Python 2.7	A script to perform fast SQL-type join operations on the corpus and compute polarity scores by quarter and year.
movie-classifier.py	Python 2.7	A script to assess the accuracy of machine learning models based on the movie reviews dataset.
emotion-main-models.R	R 3.2	An R script to compute figures and empirical models used in this report.

References

1. Manning CD, Surdeanu M, Bauer J, Finkel J, Bethard SJ, McClosky D. The Stanford CoreNLP Natural Language Processing Toolkit. In: Proceedings of 52nd Annual Meeting of the Association for Computational Linguistics: System Demonstrations; 2014. p. 55–60.

2. Maas AL, Daly RE, Pham PT, Huang D, Ng AY, Potts C. Learning Word Vectors for Sentiment Analysis. In: Proceedings of the 49th Annual Meeting of the Association for Computational Linguistics: Human Language Technologies - Volume 1 (HLT11). Stroudsburg, PA, USA: Association for Computational Linguistics; 2011. p. 142–150.

3. McAuley JJ, Leskovec J. From Amateurs to Connoisseurs: Modeling the Evolution of User Expertise Through Online Reviews. In: Proceedings of the 22nd International Conference on World Wide Web. WWW '13. New York, NY, USA: ACM; 2013. p. 897–908.

4. Toda HY, Yamamoto T. Statistical Inferences in Vector Autoregressions with Possibly Integrated Processes. Journal of Econometrics. 1995;66(1-2):225–250.

5. Hills S, Thomas R. The UK Recession in Context—What Do Three Centuries of Data Tell Us? In: Research and analysis: The UK recession in context. The Bank of England Quarterly Bulletin 2010 Q4; 2014. p. 277–291.
